# Logopenic variant of primary progressive aphasia in a bilingual non-Alzheimer’s disease octogenarian

**DOI:** 10.1590/1980-5764-DN-2025-0379

**Published:** 2026-01-09

**Authors:** Ícaro Araújo de Sousa, Bruno Henrique Carneiro Costa, Isabel Junqueira de Almeida, Maria Teresa Carthery-Goulart, Marcelo Houat de Brito, Matheus Miranda de Holanda, Tibor Rilho Perroco, Regina Miksian Magaldi, Sonia Maria Dozzi Brucki

**Affiliations:** 1 Universidade de São Paulo, Faculdade de Medicina de Ribeirão Preto, Departamento de Neurociências e Ciências do Comportamento, Ribeirão Preto SP, Brazil.; 2 Universidade de São Paulo, Faculdade de Medicina, Departamento de Neurologia, Centro de Referência em Distúrbios Cognitivos, São Paulo SP, Brazil.; 3 Faculty of Education, The University of Hong Kong, Human communication, learning, and development (HCLD), Hong Kong SAR, China.

**Keywords:** Primary Progressive Aphasia, Alzheimer Disease, Tauopathies, Afasia Progressiva Primária, Doença De Alzheimer, Tauopatias

## Abstract

A highly educated and intellectually active 84-year-old male presented with word-finding pauses and impaired sentence repetition, with preserved single-word comprehension, reading, and writing, a clinical profile consistent with the logopenic variant of primary progressive aphasia (lvPPA). Interestingly, the initial symptom was difficulty with auditory verbal comprehension in Portuguese, his second language, while comprehension in English remained initially preserved. Structural and functional imaging revealed no atrophy in the temporoparietal region or cortical hypometabolism. Plasma biomarkers, including Aβ42/40 and plasma-measured tau phosphorylated at threonine 217 (p-tau217), were within normal limits, arguing against biological Alzheimer’s disease (AD). This case illustrates a rare constellation of findings — bilingual asymmetry, negative AD biomarkers, and unremarkable neuroimaging — suggestive of a non-Alzheimer’s pathology. Alternative etiologies such as primary age-related tauopathy (PART) and argyrophilic grain disease (AGD) are discussed, emphasizing the utility of fluid biomarkers in distinguishing phenocopies within the lvPPA spectrum.

## RESUMO.

Um octogenário com alta escolaridade e intelectualmente ativo apresentou pausas na fala e prejuízo na repetição de sentenças, com preservação da compreensão de palavras isoladas, leitura e escrita, perfil clínico compatível com a variante logopênica da afasia progressiva primária (lvPPA). Interessantemente, o sintoma inicial foi déficit na compreensão auditiva em português, sua segunda língua, enquanto a compreensão do inglês permanecia preservada. Exames de imagem estrutural e funcional não revelaram atrofia temporoparietal nem hipometabolismo cortical. Biomarcadores plasmáticos, incluindo Aβ42/40 e p-tau217, estavam de acordo com os limites da normalidade, o que torna a etiologia por doença de Alzheimer improvável. Este caso ilustra uma combinação rara de achados — assimetria bilíngue, biomarcadores negativos para DA e neuroimagem relativamente preservada — sugestiva de uma patologia não Alzheimer. Consideram-se como etiologias alternativas a taupatia relacionada à idade (PART) e a doença dos grãos argirófilos (AGD), ressaltando o valor dos biomarcadores na distinção de fenótipos clínicos que mimetizam a lvPPA.

## INTRODUCTION

 Primary progressive aphasia (PPA) is a neurodegenerative syndrome characterized by a gradual, progressive decline in language, its central and defining feature. Though initially limited to language, it may later involve other cognitive domains, such as executive function, memory, and behavior^1^. 

 Historical accounts of syndromes consistent with PPA can be traced back to the late 19th century. In 1892, Arnold Pick described a case of progressive aphasia associated with circumscribed cerebral atrophy, an observation widely regarded as one of the earliest descriptions of this condition^2^. The following year, Paul Sérieux reported a case of pure verbal deafness^3^. Later in that same decade, Joseph Dejerine and Sérieux documented a 47-year-old patient who initially presented with isolated verbal deafness in the absence of other cognitive impairments. Remarkably, this condition progressed over 5 years to a clinical picture consistent with Wernicke’s aphasia^4^. However, it was not until 1982 that PPA was formally recognized as a distinct clinical entity. In a landmark publication, Marsel Mesulam described a series of six patients with slowly progressive aphasia occurring in the absence of broader cognitive or behavioral disturbances^5^. This work not only established the foundation for conceptualizing PPA as a unique neurodegenerative syndrome but also paved the way for future research and the development of formal diagnostic criteria and classification. 

 The term "logopenic" was first introduced by Mesulam in 1992 to describe patients who exhibited anomia and word-finding difficulties, while comprehension and grammar remained relatively preserved^6^. This clinical profile evolved into the definition of the logopenic variant of PPA (lvPPA), now marked by fluent speech, impaired sentence repetition, and sentence-level comprehension difficulties, despite preserved single-word comprehension. Patients often pause due to word-finding issues and show phonemic paraphasias. Research links these features to phonological loop dysfunction, a key element of working memory. Neuropathological studies have shown Alzheimer’s disease (AD) as the most common underlying pathology in lvPPA^7^. 

 Thus, this report aims to describe a patient in his eighties who developed an isolated lvPPA syndrome, with a diagnostic workup negative for AD pathology. 

## CASE REPORT

 An 84-year-old male was referred for the evaluation of progressive language decline. A retired STEM professor with over 30 years of education, he was born in China and moved to the U.S. at age 10, receiving most of his education in English and later becoming fluent in Portuguese. After emigration, he no longer used Chinese. At 29, he moved to Brazil, working as a university professor and researcher for over 30 years, and had been retired for about 10 years. His medical history included chronic hypertension and a myocardial infarction 20 years ago, treated with coronary bypass. Family history was unremarkable. 

 The first symptoms appeared about 2 years before the evaluation when he began reporting bilateral hearing difficulties. He was evaluated by an otorhinolaryngologist, and the symptoms were attributed to presbycusis. Audiometry showed moderate sensorineural hearing loss. However, there was no significant improvement with hearing aids. Notably, he initially noticed difficulty specifically in understanding Portuguese, which, about a year later, extended to English. In parallel, he began experiencing reduced attention span (subjective difficulty focusing while reading or writing) and reported minor driving accidents (frequent minor collisions or scrapes). 

 Approximately 1 year before the evaluation, he began struggling to write a book, stating he "couldn’t find the right words," though he could read normally. These word-finding difficulties gradually became noticeable in spontaneous speech. More recently, he also reported mild trouble with mental calculations. He explicitly denied memory loss and visuospatial or visuoperceptual deficits. He also denied behavioral, neuropsychiatric, or mood symptoms, though he expressed fear of not finishing his book. 

### Cognitive assessment and neurological examination

 On cognitive screening, the patient scored 28 on the Mini-Mental State Examination (MMSE) and 24 on the Montreal Cognitive Assessment (MoCA), values consistent with his educational level, though some errors were observed (Table 1). During the evaluation, he showed clear difficulties with verbal comprehension, often needing speech to be clearly enunciated and slowly delivered to be understood. In contrast, his reading and writing remained preserved. 

**Table 1:** Cognitive evaluation summary in Portuguese language

Cognitive domain	Summary of findings
Cognitive screening tests	MMSE*: 28/30 (-1 recall, -1 sentence repetition); MoCA†: 24/30 (-1 cube copy, -1 naming (rhinoceros), -1 attention, -2 repetition, -1 fluency)
Attention and executive function	Digit Span Forward: 4; Backward: 4; Clock Drawing Test (Sunderland): 10; Semantic Fluency: 15; Phonemic Fluency: 6; Intact abstraction
Memory	Figure Memory Test (BCSB‡): 10/4/7/9 – delayed recall: 7/10
Language	Mild reduction in verbal fluency with word-finding pauses; no agrammatism or apraxia of speech; intact naming (ACE-R§)’; impaired sentence repetition; preserved reading and writing
Praxia	No ideomotor (transitive/intransitive), ideational, or buccofacial apraxia
Visuospatial function	Correct overlapping pentagons; incomplete cube drawing; no dressing apraxia; no Balint’s syndrome or hemispatial neglect signs
Visuoperceptual function	No object agnosia, prosopagnosia, or color agnosia

Notes: *Mini-Mental State Examination; †Montreal Cognitive Assessment; ‡Brief Cognitive Screening Battery; §Addenbrooke’s Cognitive Examination-Revised.

 Verbal fluency was mildly impaired, with frequent word-finding pauses. There was no evidence of agrammatism or speech apraxia. Despite hesitations, he correctly named all figures on the Addenbrooke’s Cognitive Examination-Revised (ACE-R), though he had notable difficulty naming the rhinoceros. A summary of his cognitive screening results appears in Table 1. Full ACE-R results are in the supplementary material. Furthermore, neurological examination was unremarkable. 

 A comprehensive speech and language evaluation was conducted in both languages. The Portuguese assessment revealed impaired fluency and oral comprehension, especially for long sentences, along with mildly reduced sentence repetition. The English evaluation also showed impaired verbal fluency and slight naming difficulty (Table 2). Overall, the findings indicate a mild, asymmetric language impairment, predominantly affecting the later-acquired language (Portuguese), with deficits in auditory–verbal comprehension and verbal fluency. 

**Table 2:** Language assessment results in Portuguese and English.

Language tests		Results
Western Aphasia Battery revised (Brazilian version)*		
	Aphasia quotient	95.8/100
	Spontaneous speech total	20/20
	Auditory verbal comprehension total	10/10
	Repetition total	8.8/10
	Naming and word finding total	9.1/10
	Reading total	18.4/20
	Writing total	19/20
Montreal-Toulouse Language Assessment Battery (Brazilian version)+		
	Oral sentence comprehension	14/14
	Written sentence comprehension	8/8
Token test-short (Brazilian version)†		
	1st part	7/7
	2nd part	4/4
	3rd part	4/4
	4th part	4/4
	5th part	1/4
	6th part	10/13
Boston Naming Test‡		
	Portuguese version	52/60
	English version	53/60
Phonemic fluency 1’§		
	Portuguese – S	9
	English – P	9
Semantic Fluency 1’§		
	Portuguese – animals	11
	English – animals	10

Notes: *In Portuguese, the patient performed within normal limits on the Western Aphasia Battery-Revised (WAB-R), with preserved spontaneous speech, comprehension, naming, reading, and writing; mild impairment was noted in repetition; †The MontrealToulouse Battery indicated preserved sentence comprehension, while the Brazilian short Token Test showed marked deficits in parts 5 and 6, suggesting selective impairment in auditory comprehension of complex verbal commands. This aligned with observed difficulty in understanding longer spoken sentences, even with low syntactic complexity; ‡The Boston Naming Test revealed comparable performance in Portuguese and English, with mild word-finding difficulty; §Verbal fluency (phonemic and semantic) was reduced in both languages. On the Addenbrooke’s Cognitive Examination-Revised (ACE-R), impairment was restricted to fluency tasks, word and sentence repetition (supplementary material). English language comprehension through audiovisual material with subtitles was preserved. Furthermore, because the fluency tasks were administered within the ACE-R and normative data for single-letter phonemic fluency are lacking, normative scores were not included.

### Brain imaging and biomarkers

**Figure 1:**
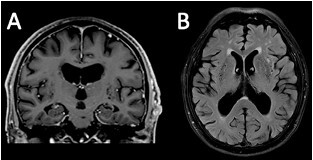
Magnetic resonance imaging (MRI). Coronal T1-weighted image shows no focal cortical atrophy, with bilateral medial temporal atrophy (MTA) score of 2 and ERICA score of 1, consistent with age-related changes. Axial T2-FLAIR image reveals marked white matter hyperintensities, classified as grade 3 on the Fazekas scale, and enlarged perivascular spaces in the basal ganglia and corona radiata, raising the differential of chronic lacunar lesions, suggesting associated microangiopathy.

**Figure 2:**
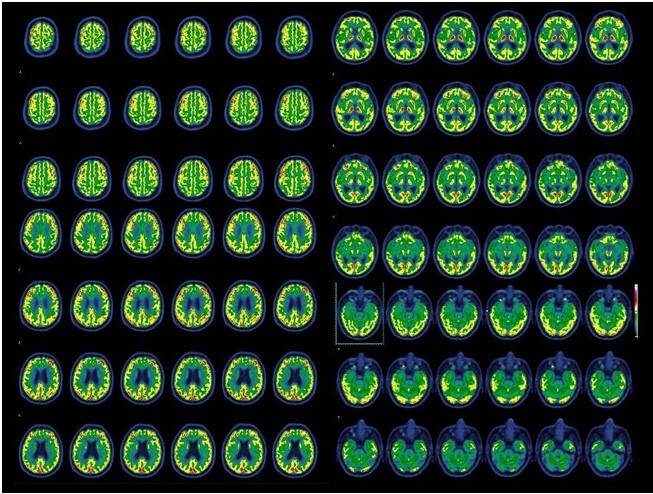
Fluorodeoxyglucose positron emission tomography-computed tomography (FDG PET-CT). PET-FDG imaging demonstrates preserved metabolic activity in the temporal and parietal cortices, with mild hypometabolism observed in the cerebellum.

 Magnetic resonance imaging (MRI) at symptom onset showed a lacunar infarct in the *basis pontis* and signs of chronic microangiopathy, including white matter hyperintensities and enlarged perivascular spaces in the basal ganglia (Figure 1). A follow-up MRI 18 months later showed no significant progression, with global atrophy appropriate for age. Positron emission tomography-computed tomography (PET-CT) revealed mild cerebellar hypometabolism, without other significant abnormalities (Figure 2). 

 Blood-based biomarker analysis revealed normal Aβ42/40 (value=0.106; reference>0.095) and p%tau217 ratios. The Amyloid Probability Score-2 (APS-2) was 5, within the normal reference range (0–47). 

## DISCUSSION

 We report the case of an octogenarian with a clinical presentation consistent with the lvPPA, characterized by word-finding pauses and impaired sentence repetition, with preserved single-word comprehension, reading, and writing. Despite this characteristic phenotype, neuroimaging revealed no temporoparietal atrophy or cortical hypometabolism, and plasma biomarkers for Aβ42/40 and p%-tau217 were within normal limits, arguing against AD. While similar cases have been reported^2-5^, the combination of bilingualism, initial auditory comprehension deficits predominantly affecting one language, advanced age, and the absence of significant structural or functional imaging abnormalities makes this case notable and warrants further discussion. 

 First, the initial symptom in this case was a selective difficulty understanding spoken Portuguese, the later-acquired language, as shown by the slight asymmetric impairment in Table 2. Such dissociations in bilingual aphasia have long been described. They may be explained by Ribot’s Law, which suggests that the most recently acquired language deteriorates before the native one^8^. However, most empirical studies suggest that language impairment in bilingual aphasics tends to be parallel, and no definitive rule governs language vulnerability across cases^8^. Notably, by the time of evaluation, the patient had also developed comprehension difficulties in English, which, though milder, suggested a progressive extension of the linguistic deficit beyond the initially affected language. 

 The patient’s auditory comprehension difficulty was prominent and became his main symptom. Initially, it could be linked to moderate sensorineural hearing loss. Interestingly, the deficit was more marked in his second language and persisted despite hearing aids and adjustments. This pattern of impaired auditory comprehension with preserved reading and writing may suggest verbal auditory agnosia. Though more often seen in stroke syndromes, this phenomenon was noted in early descriptions of PPA, as in Dejerine’s classic case of “pure verbal deafness^4,9^. It continues to be reported in association with degenerative aphasic syndromes, particularly in variants involving the phonological processing network^9^. 

 During cognitive assessment, the patient showed marked difficulty with sentence repetition, despite intact single-word comprehension and relatively preserved motor speech. This pattern is typical of phonological loop impairment, the short-term working memory system for phonemic processing and temporary verbal storage^10^. Disruption of this mechanism is regarded as a core pathophysiological hallmark of lvPPA^7^. In our patient, this was further supported by subjective difficulty with mental calculations and reduced performance on digit span and phonological verbal fluency tasks (Tables 1 and 2), which rely heavily on working memory integrity^11^. 

 Regarding the diagnostic workup, our patient did not show a reduced serum Aβ42/40 ratio, suggesting a possible alternative pathology. Although lvPPA is classically seen as an atypical form of AD, the amyloid cascade hypothesis posits that beta-amyloid accumulation is the earliest detectable event in the Alzheimer’s continuum^12,13^. Consequently, amyloid biomarkers, particularly Aβ42/40 ratio, are considered essential and early indicators in the biological diagnosis of AD^14^. Recent evidence supports the use of plasma biomarkers, like plasma-measured tau phosphorylated at threonine 217 (p-tau217) and APS2, as accurate, non-invasive tools, with diagnostic accuracies up to 90–91% and negative predictive values over 90%^15^. In this context, our patient’s normal p-tau217 levels and APS2 score of 5 strongly argue against the underlying Alzheimer’s pathology, despite a clinical phenotype consistent with lvPPA. 

 Notably, a previous study found that about one-third of lvPPA cases show a non-AD profile based on fluid biomarkers, suggesting pathological heterogeneity within this phenotype. The same study noted that lvPPA cases linked to AD tend to have greater phoneme sequencing and semantic deficits, along with a higher chance of ideomotor apraxia, features that may help distinguish them from non-AD lvPPA cases^16^. A neuropathological study of 58 PPA cases found that only 56% of lvPPA cases were due to AD, while 25% were linked to frontotemporal lobar degeneration-TAR DNA-binding protein (FTLD-TDP) and 16% to FTLD-tau, with a minority showing mixed pathologies. These results suggest that the link between lvPPA and Alzheimer’s pathology is more modest than previously indicated by in vivo amyloid imaging^17^. Notably, the study emphasized that clinical phenotype alone cannot reliably predict underlying pathology, underscoring the importance of biomarkers in etiological classification^17^. Consistent with these findings, our patient showed a prototypical lvPPA profile but lacked amyloid biomarker positivity and had no temporoparietal atrophy or cortical hypometabolism, supporting a non-AD pathological substrate. A surrogate for tau deposition could be fluorodeoxyglucose-positron emission tomography (FDG-PET), which reveals regions where neurodegeneration appears^18^. In our patient, mild hypometabolism was observed in the cerebellar hemispheres. Although cognitive cerebellar syndrome can affect language, especially phonemic more than semantic fluency, its hallmark features are dysprosody, agrammatism, anomia, and reduced oral output, often producing telegraphic speech^19^. None of these were observed in this case. 

 A recent study reported an lvPPA case with negative amyloid PET using both ^11^C-Pittsburgh compound-B and ^18^F-florbetaben, while ^18^F-florzolotau PET showed tau accumulation, suggesting a non-Alzheimer tauopathy. Although a definitive diagnosis based on PET alone remains speculative, the authors proposed primary age-related tauopathy (PART) as a possible underlying pathology^20^. PART is a pathological diagnosis in individuals with neurofibrillary tangles of 3- and 4-repeat phosphorylated tau, without significant neuritic plaques (Thal phase 0–2). Affected individuals are typically older and show mild or no amnestic impairment, unlike our case. This may be due to tau deposition in PART being usually confined to the medial temporal lobe, sparing the isocortex, which includes key language and gnosis areas (Braak stages ≤IV)^21^. Interestingly, recent data have shown that decreased semantic language is correlated with temporal lobe atrophy in patients with PART^22^. 

 Another possible underlying pathology in our case is argyrophilic grain disease (AGD), a primary tauopathy with a heterogeneous clinical spectrum. AGD may present as mild cognitive impairment or primarily with neuropsychiatric symptoms such as depression, anxiety, schizophrenia, or bipolar disorder^23^. Although AGD also typically shows an early predilection for medial temporal lobe structures, it may, in some cases, extend more diffusely into the isocortex^24^. In rare cases, AGD has been identified as the neuropathological substrate in patients with behavioral variant frontotemporal dementia. However, no cases with a language-predominant presentation have been documented to date. 

 The main limitation in our case is the lack of a biomarker confirming phosphorylated tau as the sole cause of the clinical syndrome. This reflects a broader issue: limited access to biomarkers like tau-PET in routine practice. Moreover, although less invasive and more affordable, the predictive value of serum biomarkers for AD versus cerebrospinal fluid (CSF) assays remains not fully validated^25,26^. Therefore, complementary testing with more established methods, such as amyloid-PET or CSF Aβ42/40 measurement, would have strengthened the evidence against Alzheimer’s pathology. 

 This case highlights the limited sensitivity of clinical diagnostic criteria in predicting the underlying pathology of neurodegenerative diseases, which often require postmortem confirmation. Clinical syndromes like lvPPA, though classically linked to a specific proteinopathy, may result from alternative pathologies. In the era of monoclonal antibody therapies, accurate antemortem identification of the proteinopathy, via laboratory and imaging biomarkers, is crucial to ensure appropriate treatment and clinical trial enrollment. 

## Data Availability

The datasets generated and/or analyzed during the current study are not publicly available due to ethical and privacy restrictions but are available from the corresponding author upon reasonable request.
